# Enzyme-Site Blocking Combined with Optimization of Molecular Docking for Efficient Discovery of Potential Tyrosinase Specific Inhibitors from *Puerariae lobatae* Radix

**DOI:** 10.3390/molecules23102612

**Published:** 2018-10-11

**Authors:** Haichun Liu, Yitian Zhu, Ting Wang, Jin Qi, Xuming Liu

**Affiliations:** 1 Jiangsu key Laboratory of TCM Evaluation and Translational Research, School of Traditional Chinese Pharmacy, China Pharmaceutical University, Nanjing 211198, China; tuna888@126.com (H.L.); amberzyt@outlook.com (Y.Z.); wangting5_1991@163.com (T.W.); 2 School of Science, China Pharmaceutical University, Nanjing 211198, China; 3 School of Life Science and Technology, China Pharmaceutical University, Nanjing 211198, China

**Keywords:** affinity-ultrafiltration-MS, molecular docking, tyrosinase inhibitors

## Abstract

Enzyme inhibitors from natural products are becoming an attractive target for drug discovery and development; however, separating enzyme inhibitors from natural-product extracts is highly complex. In this study, we developed a strategy based on tyrosinase-site blocking ultrafiltration integrated with HPLC-QTOF-MS/MS and optimized molecular docking to screen tyrosinase inhibitors from *Puerariae lobatae* Radix extract. Under optimized ultrafiltration parameters, we previously used kojic acid, a known tyrosinase inhibitor, to block the tyrosinase active site in order to eliminate false-positive results. Using this strategy, puerarin, mirificin, daidzin and genistinc were successfully identified as potential ligands, and after systematic evaluation by several docking programs, the rank of the identified compounds predicted by computational docking was puerarin > mirificin > kojic acid > daidzin ≈ genistin, which agreed with the results of tyrosinase-inhibition assays. Structure-activity relationships indicated that C-glycosides showed better tyrosinase inhibition as compared with O-glycosides, with reduced inhibition achieved through the addition of glycosyl, which provides ideas about the screen of leading compounds and structural modification.

## 1. Introduction

Tyrosinase (TYR) plays a vital role in melanogenesis by catalyzing the hydroxylation of l-tyrosine to l-3,4-dihydroxyphenylalanine (l-DOPA), followed by l-DOPA oxidation to DOPA quinone and phaeomelanin produced from DOPA quinone [1]; however, TYR overexpression leads to various dermatological disorders [2]. TYR inhibitors are widely used in clinical treatment for hyperpigmentation [3], in the cosmetics industry for skin whitening [4], in the food industry as anti-browning agents [5] and in the agriculture industry as insecticides [6]. However, toxicological studies showed that some existing TYR inhibitors, especially synthesized compounds have undesirable side effects, including cytotoxicity, skin cancer and dermatitis. With increasing worldwide attention on TYR inhibitors, the natural organism wins more and more attention and exportation as well, such as glabridine from glycyrrhiza and nobiletin from citrus peels. These compounds can be divided into chalcones and flavanone, coumarin derivatives, thiourea derivatives and stilbenes [7,8]. But some drawbacks limited their practical application, for example, azelaic acid shows poorly solubility; l-ascorbic acid is chemically unstable [7,9]. Thus, investigation of natural inhibitors exhibiting high activity and low side effects is urgently needed.

*Puerariae lobatae* Radix (PLR), the root of *Puerariae lobatae* (Wild.) Ohwi is widely distributed in China as antifebrile and antidiarrheal that promotes eruption and secretion [8,10]. Moreover, PLR has been used as a functional food, as well as an herbal medicine, for thousands of years. Pharmacological studies revealed that PLR exhibits skin-whitening effects for external use [11] and correlational research showed that PLR extract shows TYR inhibition [12].

Enzymes are recognized as an important target of inhibitors in drug discovery and development and there emerged many new methods to select ligands. Among these emerging ligand-searching strategies, affinity ultrafiltration (AUF)-liquid chromatography mass spectrometry (LC-MS) is widely used to screen potential molecules from nature-product extracts. The concept is based on the specific binding between target proteins and ligands [13] that allows screening according to a molecular weight cut-off for separation [14]. The advantages of UF include no need for enzyme immobilization and a simplified process that enables rapid detection and identification of enzyme-binding molecules comparing to bioassay-guided fraction [15]. However, the method has limited resolutions due to false-positive results caused by non-specific binding of molecules to non-functional sites of the enzymes or the UF membrane [16]. For this reason, many studies [17,18] introduced known ligands to block the active site of enzymes as control experiments; however, this strategy still cannot determine TYR inhibition of the selected compounds, especially given the existence of high-affinity but inefficient compounds. Molecular-docking in silico allow visualization of structural conformations and rational prediction of inhibitor affinity, rendering it a powerful technique in drug discovery. Because a variety of docking programs, including AutoDock, MOE and Glide, a comprehensive understanding of the advantages and limitations of each program would be valuable in order to enable more effective docking-based virtual screening of promising ligands [19].

Here, we proposed a strategy comprising TYR-site blocking strategy, AUF-high-performance (HPLC)-quantum time-of-flight (QTOF)-tandem MS (MS/MS) and molecular docking that improved upon the performance of the four docking tools, to clarify the effect of PLR on tyrosinase and identify the effective constituents.

## 2. Results and Discussions

### 2.1. TYR-Inhibitory Activity of the P. lobatae Radix Extract

The PLR extract showed the highest TYR inhibition rate of 45 ± 0.75% at a concentration equivalent to 2.5 mg crude medicinal herbs per milliliter, indicating that PLR capably inhibited TYR activity.

### 2.2. Optimization of UF Screening Parameters

**Figure 1:**
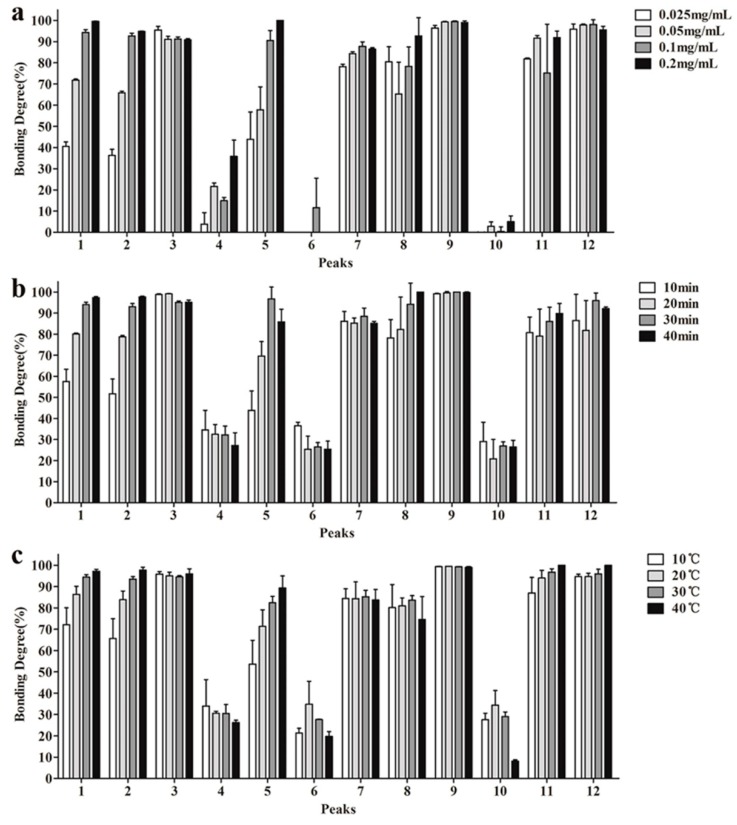

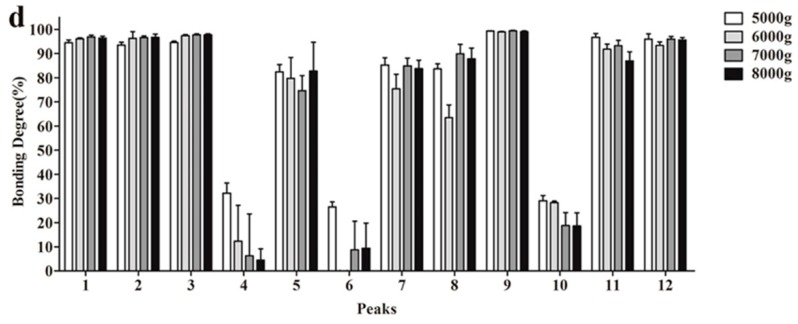
Optimization of tyrosinase (TYR) ultrafiltration parameters: (**a**) TYR concentration (0.025, 0.05, 0.1, 0.2 mg/mL); (**b**) incubation time (10, 20, 30, 40 min); (**c**) incubation temperature (10, 20, 30, 40 °C); (**d**) centrifugal force (5000×, 6000×, 7000×, 8000× *g*).

The UF parameters including TYR concentration, incubation time, incubation temperature and centrifugation speed were optimized to improve the total binding affinity and reduce background noise. By analyzing binding degrees (BDs) of filtrates according to lipid chromatographic peaks (Figure 1).

First, different TYR concentrations (0.2, 0.1, 0.05 and 0.025 mg/mL) were respectively incubated with the PLR-extract solution. HPLC results (Figure 1a) showed that most ligands exhibited increased BDs along with increased TYR concentration. In the case of insufficient tyrosinase, false-negative results could be caused by neglecting weakly binding ligands while ligands would competitively bind to the limit available TYR active sites. Therefore, an appropriate excess of tyrosinase is beneficial to ensure all potential ligands have opportunities to bind to TYR. At TYR concentrations of 0.2 mg/mL, most ligands displayed the highest BDs, whereas some peaks showed decreasing BD values compared with 0.1 mg/mL TYR. Based on these results, we selected 0.1 mg/mL for subsequent assays.

Figure 1b shows the effect of incubation time to BDs, which indicated that sufficient time is necessary to enzyme-ligand binding. After considering time-effect relationship, we selected 30 min for the further experiments.

The BDs of each component after incubation at different temperatures (10, 20, 30 and 40 °C) are shown in Figure 1c. The effect of temperature on enzyme activity is complicated, enzymatic activity usually increases along elevated temperature and increased thermal denaturation [20]. Our results showed that except compounds 4, 6 and 10, all other ligands exhibited raised BDs along with increases in temperature. Therefore, we selected 30 °C as the incubation temperature.

Figure 1d shows the relationship between BDs and centrifugation speed. Compounds **1**, **2**, **3** and **9** were less affected by this parameter, whereas the other compounds displayed their best BDs at the lowest speed. Many ligand-enzyme complexes are connected by weak bonds that are easily broken by external factors. Therefore, a centrifugation speed of 5000× *g* was sufficient to separate the complexes.

### 2.3. Screening of TYR Inhibitors from PLR Extract by UF-HPLC

**Figure 2:**
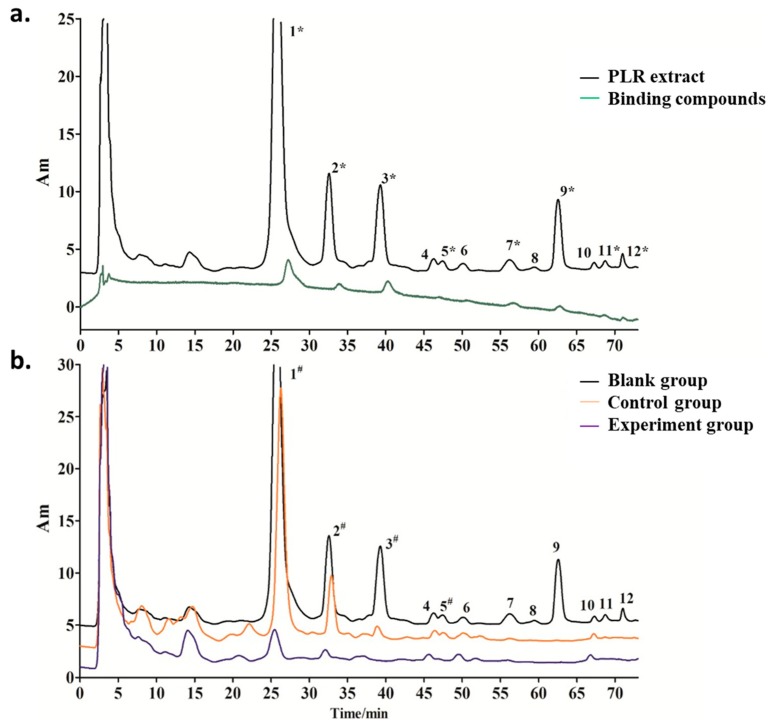
HPLC analysis: (**a**) HPLC chromatogram of Puerariae lobatae Radix extract (black) and the filtrates from test groups (green): the decrease peaks of test groups comparing with PLR extract indicate the binding compounds; (**b**) HPLC chromatogram of the filtrates respectively collected from blank groups (black), control groups (orange) and experiment groups (purple).

As showed in Figure 2, there were 12 compounds detected in PLR extract (Figure 2a). After ultrafiltration screening, because the candidate ligands retained in the chamber that leaded the corresponding peaks decrease comparing to blank group. In control groups, the application of kojic acid to block the active site of TYR, thus only these compounds that non-specific binding to the ultrafiltration membrane and other TYR sites were retained in the chamber and show decrease peak. According above principle, when (A_b_ − A_e_)/A_b_ ≥ 50%, the corresponding compounds showed binding force with TYR or UF membrane, meanwhile, these compounds could be designated as specific inhibitors that capable of binding to the TYR active site when it also meets (A_c_ − A_e_)/A_c_ ≥ 50% (where A_b_, A_c_ and A_e_ represent the peak areas of identical compounds in the blank, control and experiment groups).

Following the above criteria, there can be divided into three situations. Firstly, when (A_b_ − A_e_)/A_b_ ≥ 50% and (A_c_ − A_e_)/A_c_ ≥ 50%, the corresponding compounds represent candidate ligands. Figure 2b shows decreases in the peaks of compounds **1**, **2**, **3** and **5** (≥50%) in the three groups, suggesting these compounds as potential specific TYR ligands. Secondly, when (A_b_ − A_e_)/A_b_ < 50% and (A_c_ − A_e_)/A_c_ < 50%, the corresponding compounds do not bind to TYR. Figure 2b shows that the peak areas of compounds **4**, **6** and **10** were stable in all three groups, suggesting that they did not bind to TYR. Thirdly, compounds **7**, **9**, **11** and **12** were not recognized as specific TYR inhibitors when (A_b_ − A_e_)/A_b_ ≥ 50% but (A_c_ − A_e_)/A_c_ < 50%, suggesting non-specific binding to TYR or the UF membrane.

### 2.4. HPLC-Q-TOF MS/MS to Identify the Selected Compounds

**Figure 3:**
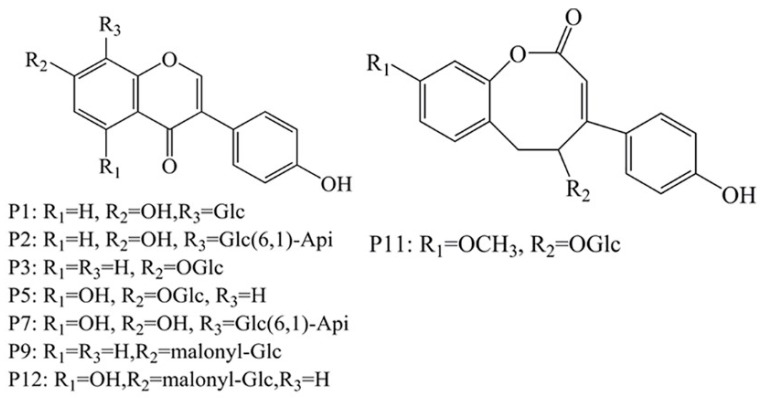
Chemical structures of puerarin (P1), mirificin (P2), daidzin (P3), genistin (P5), genistein-8-C-apiosyl (16)-Glucoside (P7), malonyl-daidzin (P9), sophoroside A and malonyl-genistin (P12).

After routine UF screening, organic reagent was added to disrupt the ligand-enzyme complexes and promote the release of ligands for HPLC-Q-TOF MS/MS analysis. By comparing the maximum UV absorption wavelength and retention time with MS data for the reference standards and previous studies [21,22,23,24,25,26,27], we found that peaks 1, 2, 3, 5, 7, 9, 11 and 12 corresponded to puerarin, mirificin, daidzin, genistin, genistein-8-C-apiosyl(16)-glucoside, malonyl-daidzin, sophoroside A and malonyl-genistin, respectively. The outputs in positive- and negative-ionization modes are shown in Table S1 and the structural representation is shown in Figure 3.

### 2.5. Comparison of Four Docking Program

The aim of the molecular docking was to predict the binding and binding interactions. Docking protocols were validated by cognate-docking with four docking protocols such as Glide, Gold, Libdock and Cdocker. If the ligand is not in the right binding mode, it would be very difficult and impossible to obtain accurate prediction results. The ligand was re-docked into the native protein by self-docking. Additionally, analysis of top 3 docked poses of ligands generated by dock programs was carried out. RMSD values lower than or equal to 2.0 Å for cognate-docking were considered to be the successful forecasting of the experimental binding mode. The RMSD values between experimental binding mode and top 3 ranked docking pose were calculated in Table 1.

The native ligands were docked to the crystal structures with remarkable accuracy. The different rates of correct pose reproduction were achieved for individual different docking methods. As shown in Table 1, Glide docking had the highest accuracy (average-RMSD 1.3404 Å). There are only minor differences (RMSD of 1.24 Å) between the best docking ligand pose (good docking score) and the co-crystal ligand conformation, suggesting high reliability of the Glide in reproducing the experimental binding mode of Mushroom Tyrosinase inhibitors. Therefore, Glide was chosen to search the Mushroom Tyrosinase binding conformations for other compounds.

### 2.6. Investigation of Enzyme-Ligand Interactions by Molecular Docking

Molecular docking study was carried out in order to explore the interaction mechanism, investigate suitable binding modes and align the dataset compounds. After Comparison of four docking program, Glide 5.5 was selected as the molecular docking tool to search the Mushroom Tyrosinase binding conformations for other compounds as it has shown good reproducibility of co-crystallized ligand conformations and accuracy in molecular docking and scoring. And the results show in Table 2. Puerarin and Mirificin showed higher score and lower energy than the standard tyrosinase inhibitors.

**Figure 4:**
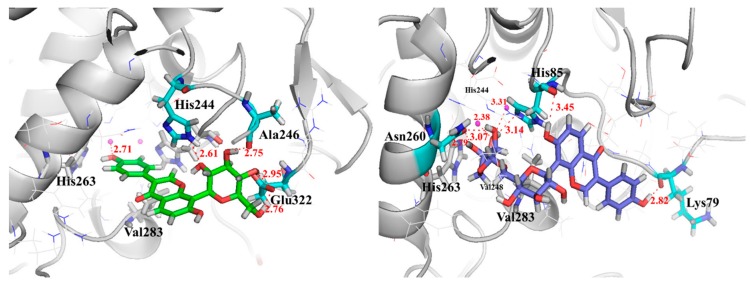
The conformations of puerarin (**left**) and mirificin (**right**) binding to the active site of TYR.

Some most potent compounds 001, 002 were selected, which were docking to the active site as a representative of the data set for in-depth analysis. They displayed significant protein ligand interactions with the key residues in Figure 4. Figure 4 displays the docking pose of inhibitor 001 and 002 (green) within catalytic active pocket of Mushroom Tyrosinase consisting of HIS61, HIS85, HIS94; HIS259, HIS263 and HIS296, Val283, ASN260, GLU256, ARG268, GLU322, TRP227, PHE264, MET257; Mushroom Tyrosinase. Compounds are projected in a cavity forming π–π inter-action with the crucial residue of His263, cation-π interaction with His244 and Van der Waals interaction with Val283. Inhibitor001 establishes the hydrogen bonds contacting with the crucial residue HIS244 and GLU322 backbone, 002 engages profitable hydrogen bond with ASN260, HIS85, LYS79. Furthermore, the proposed binding is characterized by a lipophilic sandwich of compounds between the residues of Val248 and Val283.

### 2.7. Validation of TYR Inhibitory Activity

The inhibitory activities of the four candidate PLR compounds were measured spectrophotometrically (Table 2). Ranking of their inhibitory activities is as follows: puerarin > mirificin > Kojic acid > daidzin ≈ genistin. Daidzin and genistin showed the weakest inhibition, with IC_50_ values up to 500 μM. These results agreed with molecular-docking predictions. The weak inhibition by daidzin and genistin indicated that the site-blocking strategy is limited, in that it cannot eliminate ligands exhibiting strong binding affinity but weak inhibitory effect. However, this method can eliminate compounds that bind to sites outside of the active site and simplify the subsequent docking process. Therefore, our strategy accurately and comprehensively screened high-quality TYR inhibitors from nature product and can be effectively used for drug development.

The four ligands identified here include flavonoids, although their inhibition profiles and solubility differ significantly. Daidzin and genistin are classified as *O*-glycosides, whereas puerarin and mirificin are both C-glycosides. According to their ability to inhibit TYR, we speculated that C-glycosides are more effective TYR inhibitors than *O*-glycosides, although this requires further study for validation. Puerarin and mirificin share a similar structural framework but mirificin has additional glycosyl moieties. According to previous studies [21,28,29], the oxidation-inhibitory activities of flavonoids are negative correlated with the amount of glycosyl present, with molecular-docking analysis also indicating that the addition of glycosyl reduced TYR inhibition due to steric hindrance and changes in polarity. Based on these results, glycosyl analogically affected TYR inhibition by these ligands.

## 3. Materials and Methods

### 3.1. Apparatus

A semi micro balance (0.01 mg; Mettler Instrument Co., LTD., Shanghai, China), a PHS-25 pH meter (INESA Scientific Instrument Co., Ltd, Shanghai, China), a UV-2550 UV-vis spectrophotometer (Shimadzu, Kyoto, Japan), an LC-2010 HPLC system (Shimadzu) equipped with a Phenomenex Luna C_18_ analytical column (250 mm × 4.6 mm, 5 µm), a 6520 Accurate-Mass Q-TOF LC/MS system (Agilent, Santa Clara, CA, USA) and Omega Nanosep ultrafilter centrifuge with a 10-kDa molecular weight cut-off ultrafiltration membrane (Pall Corp., Port Washington, NY, USA) were obtained for this study.

### 3.2. Reagents and Materials

PLR was purchased from Nanjing Shang Yuan Tang Medicinal Store (Nanjing, China). Analytical grade methanol, potassium dihydrogen phosphate and dipotassium hydrogen phosphate were purchased from Nanjing Chemical Reagent Factory (Nanjing, China). HPLC-grade acetonitrile was purchased from Tedia (Fairfield, OH, USA). Analytical grade dimethyl sulfoxide (DMSO) was purchased from Shanghai Chemical Reagent Factory. The reference standards puerarin, daidzin and genistin were purchased from Cheng Du Must Biotechnology Co., Ltd. (purity >98%; Chengdu, China). Mirificin was purchased from Nanjing Spring & Autumn Biological Engineering Co., Ltd (purity >98%; Nanjing, China). Koic acid was purchased from Shanghai Yuanye biological technology Co., Ltd. (purity >98%; Shanghai, China). TYR and l-tyrosine were purchased from Sigma-Aldrich (St. Louis, MO, USA). Ultrapure water was prepared using a Millipore water purification system (Millipore, Bedford, MA, USA).

### 3.3. Preparation of Extraction and Stock Solutions

PLR was oven-dried, pulverized into powder and sieved (60 mesh), followed by storage in the shade before use. Powder (2 g) was accurately weighed and extracted twice by ultra-sonication for 30 min with 75% (*v*/*v*) methanol. The extracted solution was filtered, mixed and dried by rotary vaporization at 60 °C under reduced pressure. Residues were resolved using 1 mL DMSO as a stock solution and stored at 4 °C before use. The PLR-extract solution was pre-pared by diluting the stock solution with phosphate-buffered saline (PBS; pH 6.8) at a 1:50 ratio (*v*/*v*), with the solution containing 2% DMSO and 40 mg/mL equivalent raw medicine.

TYR stock solution was prepared by suspending TYR in PBS at 1 mg/mL (pH 6.8) and diluting it with PBS to desire concentration when use.

### 3.4. Investigation of the UF Parameters

The UF screening parameters including TYR concentration, incubation time, incubation temperature and centrifugation speed were investigated.

PLR-extract solution (100 µL) mixed with 100 µL diluted TYR solution and incubated at 30 °C on a shaking platform for 30 min, followed by centrifugation for 15 min at 5000× *g*. The unbound compounds separated through the ultrafiltration membrane into the filtrates. The filtrates were analyzed by HPLC, with separation performed using the Venusil MP C_18_ column (4.6 × 250 mm, 5 µm; Agela, Tianjin, China) at 30 °C. The injected volume was 20 µL and the composition of the mobile phase was 0.1% phosphoric acid (solvent A) and acetonitrile (solvent B) delivered as a gradient [0–15 min, 10–15% (B); 40–60 min, 15–20% (B); 60–70 min, 20–30% (B); and 70–85 min, 30–10% (B)]. The binding force of the compounds to TYR was defined as the binding degree (BD), which was calculated according to Equation (1).

(1) $$\mathrm{Binding}\;\mathrm{degree}\;(\%)\;=\;\frac{S_{k}-S_{e}}{S_{k}}\;\times \;100\%$$

where S_k_ and S_e_ represent the peak areas of a compound in the blank and experiment groups, respectively, in the HPLC chromatograms.

### 3.5. TYR-Affinity UF

PLR-extract solution (100 µL) mixed with 100 µL diluted TYR solution and incubated at the best UF parameters. The unbound compounds can pass through the ultrafiltration membrane into the filtrates (F1), whereas the enzyme-ligand complexes remained in the chamber. After washing the retained complexes twice with 200 µL PBS (pH 6.8) to remove the residual unbound compounds, 200 µL methanol (80% (*v*/*v*)) was added into chamber and ultrasonic for 15 min to disrupt the complexes and release the ligands. The mixture was then centrifuge at 5000× *g* for 15 min, the filtrates (F2) containing the selected compounds were passed through a 0.45 µm filter membrane prior to HPLC analysis. For blank groups, PBS (pH 6.8) containing 2% DMSO was used to incubate with the TYR solution, followed by performance of the protocol described here. All experiments were performed in triplicate.

### 3.6. TYR-Site Blocking UF Screening

TYR-site blocking UF screening was performed as control group. 50 µL TYR (0.1 mg/mL) incubated with 50 µL Kojic acid (0.05 mg/mL) for 30 min at 30 °C to block the TYR active site. The PLR extract solution was added to the reaction solution and the protocol described in Section 2.5 was followed prior to HPLC analysis. All experiments were performed in triplicate.

### 3.7. HPLC Q-TOF MS/MS Analysis

The filtrates obtained after centrifugation were analyzed by HPLC Q-TOF MS/MS using a 6520 Accurate-Mass Q-TOF LC/MS system equipping with an electrospray ionization (ESI) interface. The ESI source operated in negative mode and the operating parameters included a drying gas (N_2_) flow rate of 8.0 mL/min, atomizing pressure of 40 psig, capillary voltage of 4000 V, capillary temperature of 325 °C, collision energy ranging from 20 V to 40 V and skimmer voltage of 60 V. The mass range was set from 100 units to 1000 units. HPLC parameters were the same as those described in Section 2.4, except replace solution B with 0.1% formic acid.

### 3.8. Molecular Docking

**Figure 5:**
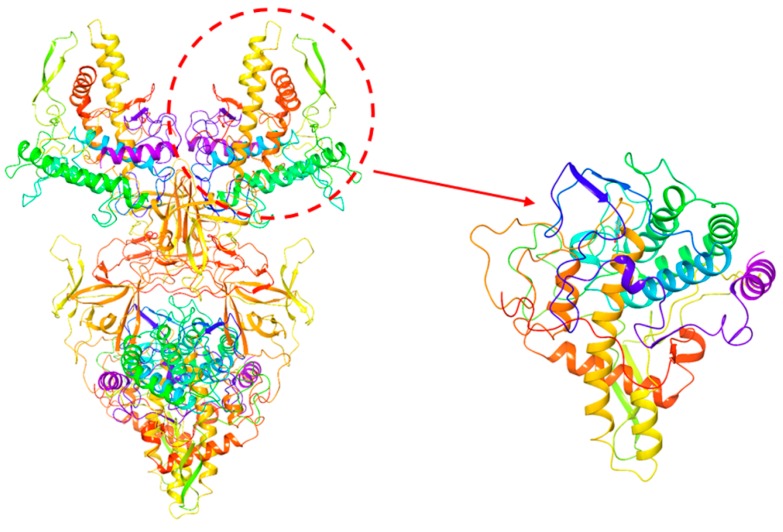
Mushroom Tyrosinase Crystal complex structure (PDB ID: 2Y9X).

The crystallized complex structure of Mushroom Tyrosinase (PDB ID: 2Y9X, Resolution 2.78 Å, Ligand OTR; Figure 5) was downloaded from RCSB protein data bank (http://www.rcsb.org/pdb/), The A-chain was used for docking study. Ligand OTR was extracted from the A-chain and then the atom types were modified, hydrogen atoms and the Gasteiger-Hückel charges were added within Sybyl 6.9 [30]. 3D structures of the other compounds were constructed based on modifying Ligand OTR using the Sketch module of the Sybyl software package. The Gasteiger-Huckel partial atomic charges were calculated for each constructed structure and energy minimization was performed using Powell gradient algorithm with the Tripos force field and a convergent threshold of a maximum deviation of 0.01 kcal/(mol × Å). The minimized structure was used as the initial conformation for molecular modeling.

After the receptor and ligands were prepared, molecular docking was first validated by re-docking the crystal ligand to ensure that the molecular docking could recapture feasible conformation. Four docking protocols, such as Glide, Gold, Libdock and Cdocker, were compared and the best would be selected as the ultimate molecular docking tool.

Glide. Re-docking the crystal ligand (PDB ID: 2Y9X) was used to evaluate Glide [31] docking to ensure that the molecular docking could recapture feasible conformation. The PDB 2Y9X selected was prepared with the Protein Preparation Wizard workflow. Then the generated receptor grid was centered on the co-crystallized ligand OTR, which was defined as the ligand-binding site search region. The compounds to be docked were confirmed by an enclosing box. Furthermore, the compound set was minimized using OPLS-2005 force field with the conformational search method in MacroModel module. The top 10 conformations of each ligand were allowed to save and ranked by docking scores. The best conformation of each compound comprised the output on the basis of the Glide-score and their binding interactions formed between the compounds and the active site. All the remaining parameters were kept as default settings. Finally, the potential compounds were flexibly docked into the binding site with standard precision (SP) docking mode. In addition, the binding interactions between the ligands and protein were showed.

Gold. Gold developed by Jones et al has been commercially released by the Cambridge Crystallographic Data Center [32]. GOLD explores the ligand conformation by a genetic algorithm (GA) search strategy. To provide the most accurate docking results, all docking calculations were performed using the default calculation mode. At the same time, early termination was turned on to accelerate the docking calculations. The scoring function of Gold_Score was selected for calculation.

Cdocker. Cdocker is an implementation of an all-atom CHARMm force field-based molecular docking tool [33]. Ligands were docked into active site of the rigid receptor with a simulated annealing method and various conformations for ligand were generated through molecular dynamics simulation. This-Cdocker inter-action energy was calculated to rank docking poses. The pose with the best-Cdocker interaction energy (-ECD) was selected.

LibDock. LibDock docking engine treats the receptor as rigid and treats ligand molecules as flexible structures [33]. LibDock docks ligands (after removing hydrogen atoms) into a binding site guided by binding hotspots. It aligns docked ligand conformations to polar and apolar receptor interactions sites, that is, hotspots. Conformations can be either pre-calculated or generated on the fly. Since some of the output poses may have hydrogen atoms in close proximity to the receptor, a CHARMm minimization step can be optionally enabled to further optimize the docked poses. The highest ranking docked conformers/poses were scored using LibDockscore.

### 3.9. TYR Inhibit Assay

TYR inhibition by PLR extract solution, selected compounds and Kojic acid was analyzed by spectrophotometry. The solution comprised 50 µL of the mixed ligand solution (the corresponding ligand standards were diluted 50-fold with PBS) incubated with 50 µL TYR solution (0.1 U/mL ) for 30 min at room temperature, followed by the addition of 100 µL l-tyrosine (0.4 mM) and incubation for another 30 min at 30 °C. Absorbance was measured in triplicate at 480 nm and inhibition ratios were calculated from the mean of the triplicate observations according to Equation (2):

(2) $$\mathrm{Inhibitory}\;\mathrm{Ratio}\;(\%)\;=\;\frac{A_{b}-A_{e}}{A_{b}}\;\times \;100\%,$$

where A_b_ and A_e_ represented the absorption of blank groups and experiment groups, respectively.

## 4. Conclusions

In our work, the site-blocking strategy employing UF-HPLC Q-TOF-MS/MS combined with optimized molecular docking was applied to screen high-quality TYR-specific inhibitors from PLR extract. The UF-HPLC-Q TOF-MS/MS can effectively separate the ligand-enzyme complexes from unbound compounds and provides the structural information of the selected compounds. Kojic acid, a TYR inhibitor, was used to distinguish the selective ligands from the non-selective compounds, which may decrease the number of false positives. Moreover, the optimized molecular docking was used to anticipate the inhibition of selected compounds and enabled identification of compounds that exhibit high binding affinity but low inhibition. In result, there were four promising TYR inhibitors (puerarin, mirificin, daidzin and genistin) were identified from PLR. This strategy can narrow the time and cut workforce. The results demonstrated the efficiency and reliability of this strategy. In future study, this strategy can be applied for screening other enzyme inhibitors and provides a useful reference for evaluating lead compounds from natural products.

## Figures and Tables

**Table 1:** RMSD of top 3 configurations obtained through docking for docking reliability validated.

Conformation	RMSD (Å)			
	Glide	Gold	Cdocker	LibDock
1	1.2840	1.6428	1.4536	3.1159
2	1.2699	1.5603	1.3451	2.9661
3	1.4672	1.6947	1.3515	2.9537

**Table 2:** The scores of molecular docking to the Mushroom Tyrosinase (PDB ID: 2Y9X) and TYR inhibitory activity of selected ligands and kojic acid.

Peak	Compound	Score	Energy	IC_50_ ± SD (μM)
1	Puerarin	−6.21	−53.34	9.83 ± 1.45
2	Mirificin	−6.04	−51.65	12.66 ± 2.77
3	Daidzin	−5.17	−40.65	>500
5	Genistin	−5.48	−42.43	>500
control group	Kojic acid	−5.87	−47.31	46.91 ± 3.21

## Supplementary Materials

The following are available online. Table S1: Retention time, maximum absorption UV wavelength and the MS data of compound **1**, **2**, **3**, **5**, **7**, **9**, **11** and **12** selected from PLR extract.
