# Supplementary material for: Enzyme-Site Blocking Combined with Optimization of Molecular Docking for Efficient Discovery of Potential Tyrosinase Specific Inhibitors from Puerariae lobatae Radix
Source: Molecules. 2018 Oct 11;23(10):2612. doi: 10.3390/molecules23102612 (PMC6222779; doi:10.3390/molecules23102612)
Supplement: Supplementary file 1 [file molecules-23-02612-s001.pdf]

Table S1. Retention time、maximum absorption UV wavelength and the MS data of compound 1, 2, 3, 5, 7, 9, 11, and 12 selected from PLR.

Peak	TR	UV (nm)	Identification	Formula	Negative ion(m/z)			Positive ion(m/z)		
					Proposal ions	MS/MS	Error (ppm)	Proposal ions	MS/MS	Error (ppm)
1	25.7	248	Puerarin	$C_{21}H_{20}O_9$	$[M-H]^-$	415.1034	0.13	$[M+H]^+$	417.1180	-1.42
		305			$[M- C_4H_8O_4-H]^-$	295.0605		$[M+H-2H_2O- C_4H_4O_2]^+$	297.0765	
					$[M- C_4H_8O_4-H_2O-H]^-$	277.0488		$[M+H-2H_2O-CH_2O-C_4H_4O_2]^+$	267.0652	
2	32.6	247	Mirificin	$C_{26}H_{28}O_{13}$	$[M-H]^-$	547.1451	1.12	$[M+H]^+$	549.1601	0.31
		306			$[M-api- C_4H_7O_3-H]^-$	295.0603		$[M+H-api]^+$	417.1138	
					$[M-api- C_4H_7O_3-CO-H]^-$	267.0653		$[M+H-api-2H_2O-CH_2O]^+$	351.0980	
								$[M+H-api-2H_2O-CH_2O-C_4H_2O_2]^+$	276.0652	
3	39.3	248	Daidzin	$C_{21}H_{20}O_9$	$[M+HCOO-H]^-$	461.1082	1.77	$[M+H]^+$	417.1182	-0.46
					$[M-H]^-$	415.1007		$[M+H-glc]_+$	255.0655	
					$[M-glc-H]^-$	253.0491				
5	47.5	206	Genistin	$C_{21}H_{20}O_{10}$	$[M+HCOO-H]^-$	477.1035	0.81	$[M+H]^+$	433.1137	-1.8
		263			$[M-glc-H]^-$	269.0331		$[M+H-glc]^+$	271.0618	
7	56.2	208	Genistein-8-C-	$C_{26}H_{28}O_{14}$	$[M-H]^-$	563.1401	0.94	$[M+H]^+$	565.1544	1.39
		244	apiosyl(1-6)-		$[M-api- C_4H_7O_3-H]^-$	310.9359				
			glucoside		$[M-api- C_4H_7O_3-CO-H]^-$	283.9540				
9	62.6	249	Malonyl-	$C_{24}H_{22}O_{12}$	$[M-H]^-$	501.1026	2.49	$[M+H]^+$	503.1183	0.20
			Daidzin					$[M+H-(mal)glc]^+$	255.0669	
11	68.7	242		$C_{24}H_{26}O_{10}$	$[M-H]^-$	473.1458	-1.01	$[M+H]^+$	475.1594	1.00
		264	Sophoroside A					$[M+H-glc]^+$	313.1073	
								$[M+H-glc-CO_2]^+$	267.2674	
12	71.0	261	Malonyl-	$C_{24}H_{22}O_{13}$	$[M-H]^-$	517.1013	-4.89	$[M+H]^+$	519.1141	-1.51
			Genistin					$[M+H-(mal)glc]^+$	271.1492	
